# Underestimation of respiratory symptoms by smokers: a thorn in chronic obstructive pulmonary disease diagnosis

**DOI:** 10.1038/s41533-021-00226-y

**Published:** 2021-03-12

**Authors:** Evdoxia Gogou, Ourania S. Kotsiou, Dimitra S. Siachpazidou, Maria Pinaka, Charalampos Varsamas, Fotini Bardaka, Irini Gerogianni, Chrysi Hatzoglou, Konstantinos I. Gourgoulianis

**Affiliations:** 1 Department of Physiology, Faculty of Medicine, University of Thessaly, BIOPOLIS, Larissa, Greece; 2 Department of Respiratory Medicine, Faculty of Medicine, University of Thessaly, BIOPOLIS, Larissa, Greece

**Keywords:** Respiratory signs and symptoms, Epidemiology

## Abstract

Primary care centers are ideal positions to identify chronic obstructive pulmonary disease (COPD). We determined the COPD prevalence among ever-smokers aged 40–65 years attending a 2-year program conducted in 22 Greek primary healthcare centers and made comparisons between genders, patients less than or greater than 55 years, and newly or previously diagnosed COPD patients. A total of 117 persons, after studying 1100 people, were diagnosed with previously unknown or known COPD, providing a COPD prevalence of 10.6% among the study population. In all, 7.5% of the participants were newly diagnosed with COPD. Women with COPD reported smoking less but experienced worse respiratory and depressive symptoms than men. A total of 19% of the COPD population below 55 years experienced wheezing and exacerbations more frequently than older patients. Newly diagnosed COPD patients were significantly younger, reported a significant burden of symptoms without seeking medical help. Primary health care has a crucial role in the early detection of COPD among unsuspecting smokers.

## Introduction

Chronic obstructive pulmonary disease (COPD) is a progressive chronic lung disease characterized by irreversible airflow obstruction^1^. COPD is already the third most common cause of adult mortality, something that the World Health Organization had not predicted to occur until 2030, and a leading cause of adult hospitalization globally^2^. Cigarette smoking is recognized as the leading cause of the disease. A total of 10–20% of smokers develop clinically significant COPD^1^. Importantly, the prevalence of COPD has increased in women, as the prevalence of smoking habit in females has grown progressively^1^.

There is a linear increase in COPD prevalence with patients’ age, suggesting that changes related to aging may contribute to COPD pathogenesis. Although COPD occurs most often in older adults, it can also affect people under 65 years old^3^. It has been reported that 44% of COPD patients are below the retirement age of 62 in the United Kingdom^4^. A subgroup of smokers appears particularly vulnerable to tobacco smoke and develops COPD in their early years^5^.

Greece has been suffering a high burden of COPD, although the reported prevalence rates are lower than expected when the high smoking rates in the country among European Union states are taken into account. An increasing COPD prevalence rate of up to 18.2% is apparent within the past 10 years in Greece^6,7^. COPD patients in Greece had a mean age between 60 and 70 years when first diagnosed according to studies that were conducted in the pre- and post-crisis periods^6–8^.

Since the radical cure is not clear for COPD, prevention is better than therapeutic management, and the screening for early diagnosis of COPD is of particular importance^9–11^. COPD causes breathlessness, manifesting initially during exertion, then it increases progressively along with the airflow obstruction and predisposes exacerbations and serious illness^2^. Therefore, distilling the essence of breathlessness is a specific responsibility of doctors, as dyspnea could be the first vital symptom of a chronic and reversible if identified and treated early enough respiratory disease^10^. Notably, several studies have revealed a high prevalence of COPD among smokers with minimal or no symptoms^9^.

A remarkable proportion of COPD patients are undiagnosed and made case finding worthwhile^12^. Primary care centers are ideal positions to identify COPD, constituting the first filter in the pathway to care for the majority of patients. The main objective of this study was to determine the COPD prevalence rate among ever-smokers in pre-retirement, aged 40–65 years, attending a 2-year primary care spirometry surveillance program in central Greece. The secondary objectives were to evaluate their demographic, clinical, and spirometric characteristics and make comparisons between patients less than or greater than 55 years of age, genders, and newly and previously diagnosed COPD patients.

## Results

**Fig. 1:**
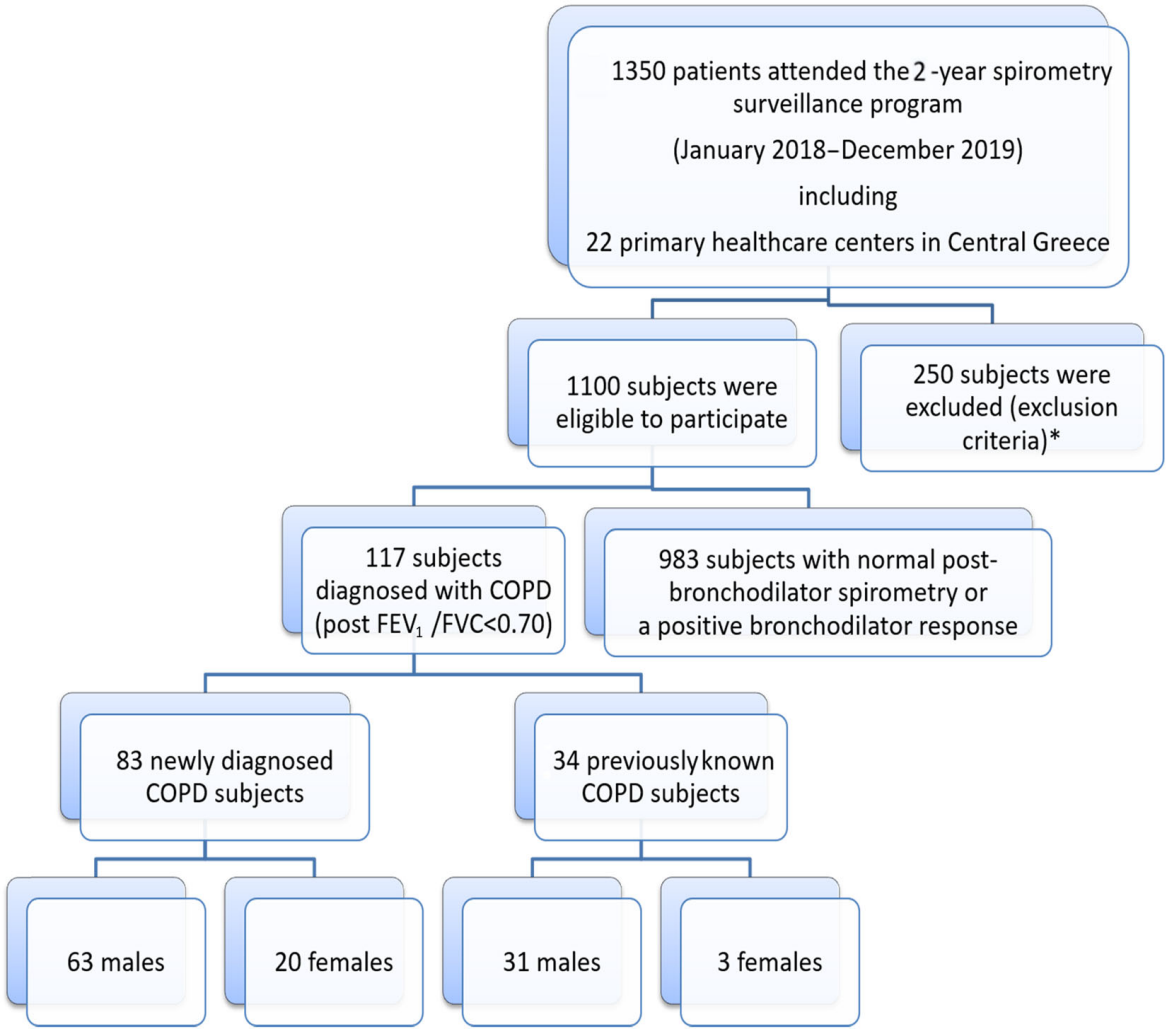
Flowchart of the study selection process. In our study, 1350 patients attended the 2-year spirometry surveillance program, from January 2018 to December 2019, in 22 Primary Health Centers in Central Greece. From all these patients, 1100 were smokers and ex-smokers aged 40–65 years old, the others 250 subjects were excluded according to the exclusion criteria of our study. From those subjects were eligible to participate, 117 subjects diagnosed with COPD (post FEV1/FVC<0.70) and 983 subjects had either normal postbronchodilator spirometry or positive bronchodilator response. Of the 117 patients diagnosed with COPD, 83 were diagnosed for the first time and 34 were previously known COPD patients. According to the gender, those with newly diagnosed COPD patients, 63 were males and 20 females, while of those with previously known COPD, 31 were males and only 3 were females. *Exclusion criteria: no smoking history, a history of lower or upper respiratory infection or antibiotic treatment within the last month, an operative procedure or acute myocardial infarction within the last three months, or inability to perform spirometry.

The flowchart of the study selection process is presented in Fig. 1. A total of 1100 smokers and ex-smokers aged 40–65 were included in the spirometry surveillance program according to inclusion criteria. A total of 117 newly diagnosed or previously known COPD patients were detected, providing a COPD prevalence of 10.6% among the study population. Of the 117 patients diagnosed with COPD after studying 1100 people, 83 (7.5%) were diagnosed for the first time, representing a rate of 70.94% of COPD underdiagnosis.

### Characteristics of all COPD patients

The demographic, clinical, and spirometric characteristics of all COPD patients and comparisons between genders are shown in Table 1. Nearly four-fifths of all COPD patients were males with a mean age of 60.3 ± 5.8 years and a mean body mass index (BMI) within the overweight range. The majority were married, current smokers, and almost half of them were high school graduates. Importantly, 89% of the COPD population mentioned at least one respiratory symptom. Dyspnea was the most commonly reported symptom, followed by cough, wheezing, and sputum. One-third of COPD patients had dyspnea score greater than or equal to 2, i.e., breathlessness on level ground (grade 2), exercise limitation (grade 3), or dyspnea on rest (grade 4) according to modified Medical Research Council (mMRC) scale, while 40.2% of COPD patients reported COPD assessment test (CAT) total score of ≥10. More than half of the patients had at least one comorbidity and one exacerbation (Table 1).

**Table 1:** Demographic, clinical, and spirometric characteristics of chronic obstructive pulmonary disease (COPD) patients (*N* = 117) and gender-associated differences.

Characteristics	Total (*n* = 117)	Males (*n* = 94)	Females (*n* = 23)	*P* values
*Age (years)*	60.3 ± 5.8	60.6 ± 5.8	59.2 ± 6.1	0.313^a^
*BMI (kg/m*^*2*^*)*	27.9 ± 5.1	28.1 ± 4.7	27.2 ± 6.6	0.443^a^
*Educational level*				0.423^b^
Elementary education	48 (41.0)	42 (44.7)	6 (26.1)	
Secondary education	60 (51.3)	45 (47.9)	15 (65.2)	
Tertiary education	9 (7.7)	7 (7.4)	2 (8.7)	
*Marital status*				0.446^b^
Not married	4 (3.4)	4 (4.3)	0 (0)	
Married	105 (89.7)	83 (88.3)	22 (95.7)	
Divorced	3 (2.0)	2 (2.1)	1 (8.7)	
Widowed	5 (4.3)	5 (5.3)	0 (0)	
*Current smokers*	88 (75.2)	69 (73.4)	19 (82.6)	0.265^b^
*Pack-years of smoking (years)*	50.8 ± 25.6	60.0 ± 24.0	26.0 ± 15.0	**<0.001**^**a**^
*Age started smoking (years)*	18.6 ± 7.1	17.3 ± 6.7	24.3 ± 5.9	**<0.001**^**a**^
*Respiratory symptoms (yes)*	104 (88.9)	84 (89.4)	20 (87.0)	0.359^b^
Cough	67 (57.3)	53 (56.4)	14 (60.9)	0.441^b^
Sputum	63 (53.8)	54 (57.4)	9 (39.1)	0.089^b^
Wheezing	66 (56.4)	53 (56.4)	13 (56.5)	0.590^b^
Dyspnea	81 (69.2)	64 (68.1)	17 (73.9)	0.393^b^
*mMRC* *≥* *2*	38 (32.5)	31 (33.0)	7 (30.4)	0.513^b^
*CAT* *≥* *10*	47 (40.2)	33 (35.1)	14 (60.9)	**0.022**^**b**^
*Exacerbations (yes)*	61 (52.2)	44 (46.8)	12 (52.2)	0.409^b^
One	49 (41.9)	39 (41.5)	10 (43.5)	0.470^b^
Two or more	7 (6.0)	5 (5.3)	2 (8.7)	0.470^b^
*Comorbidities (yes)*	70 (59.8)	57 (60.6)	13 (56.5)	0.447^b^
Hypertension	54 (46.2)	46 (48.9)	8 (34.8)	0.162^b^
Diabetes mellitus	18 (15.4)	16 (17.0)	2 (8.7)	0.261^b^
Cardiovascular disease	17 (14.6)	17 (18)	0 (0)	0.110^b^
Hyperlipidemia	37 (31.6)	31 (33.0)	6 (26.1)	0.356^b^
Depression	4 (3.4)	1 (1.1)	3 (13.0)	**0.024**^**b**^
*Newly diagnosed with COPD*	83 (70.9)	63 (67.0)	20 (87.0)	**0.046**^**b**^
*Postbronchodilator FEV*_*1*_ *(ml)*	2.2 ± 0.6	2.3 ± 0.6	1.7 ± 0.5	**<0.001**^**c**^
*Postbronchodilator FEV*_*1*_ *(%)*	73.3 ± 17.1	72.6 ± 16.5	76.3 ± 19.4	0.363^c^
*Postbronchodilator FEV*_*1*_*/FVC (%)*	60.9 ± 6.4	63.4 ± 6.2	66.0 ± 6.9	0.075^a^
*GOLD stages*				0.241^b^
Stage I	47 (40.1)	35 (37.2)	12 (52.2)	
Stage II	61 (52.1)	53 (56.4)	8 (34.8)	
Stage III	7 (6.0)	5 (5.3)	2 (8.7)	
Stage IV	2(1.7)	1 (1.1)	1 (4.3)	
*2020 GOLD groups*				0.619^b^
Group A	64 (54.7)	62 (66.0)	2 (8.7)	
Group B	45 (38.5)	26 (27.7)	19 (82.7)	
Group C	6 (5.1)	5 (5.3)	1 (4.3)	
Group D	2 (1.7)	1 (1.0)	1 (4.3)	

Data are expressed as mean ± standard deviation or as frequency (percentage). Statistically significant *P* values are presented in bold.

*BMI* body mass index, *CAT* COPD assessment test, *FEV*_*1*_ forced expiratory volume in 1 s, *FVC* forced vital capacity, *mMRC* modified Medical Research Council.

^a^Mann–Whitney *U* test.

^b^χ^2^ test.

^c^Independent-samples *t*-test.

A total of 92% of COPD patients were classified on Stages I/II. We found that 40% and 30% of COPD patients in GOLD Stage I/II scored more than 10 on the CAT score, and greater than or equal to 2 on mMRC score, respectively. COPD patients in GOLD Stage III/IV had more frequent wheezing symptoms than early-stage patients, as expected. No significant association was detected between different GOLD stages and BMI, educational level, marital status, or comorbidities. Interestingly, 54.7% of all COPD patients were classified into GOLD COPD Group A having fewer symptoms and a low risk of exacerbations, and more than one-third were classified into GOLD COPD Group B, having more symptoms and a low risk of exacerbation.

Almost 20% of all COPD patients were women. The majority of women (82.7%) diagnosed with COPD were in Group B, which means they had higher symptoms. The majority of men diagnosed with COPD were in Group A (66%), and one-third were in Group B (Table 1). Rates of newly diagnosed were higher among females compared to males (87% vs. 67%, *P* = 0.046). Women with COPD reported smoking fewer cigarettes than men, had an older age at starting smoking (24.3 ± 5.9 vs. 17.3 ± 6.7, *P* < 0.001), but experienced worse symptoms (CAT score ≥10) and had more frequent depression compared to males (Table 1).

### Comparison of COPD patients’ characteristics according to age

A total of 19% (25/117) of the COPD population were below 55 years. Comparisons of demographic, clinical, and spirometric characteristics between COPD patients over and below 55 years of age are presented in Table 2.

**Table 2:** Comparisons of demographic, clinical, and spirometric characteristics between COPD patients over and below 55 years of age.

Characteristics of COPD patients (*N* = 117)	Below 55 years (*n* = 25)	Over 55 years (*n* = 92)	*P* values
*Males*	17 (68.0)	77 (83.7)	0.075^a^
*BMI (kg/m*^*2*^*)*	28.3 ± 7.1	27.8 ± 4.4	0.739^b^
*Educational level*			0.538^a^
Elementary education	8 (32.0)	40 (43.5)	
Secondary education	15 (60.0)	45 (48.9)	
Tertiary education	2 (8.0)	7 (7.6)	
*Marital status*			0.246^a^
Not married	2 (8.0)	2 (2.1)	
Married	23 (92.0)	82 (89.1)	
Divorced	0 (0)	3 (3.2)	
Widowed	0 (0)	5 (5.4)	
*Current smokers*	22 (88.0)	66 (71.7)	0.075^a^
*Ex-smokers*	3 (12.0)	26 (28.3)	0.075^a^
*Pack-years of smoking (years)*	36.0 ± 19.0	55.0 ± 26.0	**<0.001**^**b**^
*Age started smoking (years)*	18.2 ± 3.6	18.8 ± 7.8	0.612^b^
*Respiratory symptoms (Yes)*	24 (96.0)	80 (87.0)	0.701a
Cough	16 (64.0)	51 (55.4)	0.297a
Sputum	15 (60)	48 (52.2)	0.320a
Wheezing	19 (76.0)	47 (51.1)	**0.021**^**a**^
Dyspnea	17 (68.0)	64 (69.6)	0.530^a^
*mMRC* *≥* *2*	6 (24.0)	32 (34.8)	0.220^a^
*CAT* *≥* *10*	10 (40.0)	37 (40.2)	0.586^a^
*Exacerbations (yes)*	17 (68.0)	39 (42.4)	**0.020**^**a**^
One	13 (52.0)	36 (39.1)	**0.014**^**a**^
Two or more	4 (16.0)	3 (3.2)	**0.014**^**a**^
*Comorbidities (yes)*	10 (40.0)	60 (67.4)	**0.021**^**a**^
Hypertension	6 (24.0)	48 (52.2)	**0.010**^**a**^
Diabetes mellitus	2 (8.0)	16 (17.4)	0.204^a^
Cardiovascular disease	0 (0)	17 (18.5)	0.110^a^
Hyperlipidemia	31 (33.0)	6 (26.1)	0.356^a^
Depression	2 (8.0)	2 (2.1)	0.120^a^
*Newly diagnosed patients*	17 (68.0)	66 (71.7)	0.446^a^
*Postbronchodilator FEV*_*1*_ *(ml)*	2.3 ± 0.9	2.2 ± 0.6	0.366^c^
*Postbronchodilator FEV*_*1*_ *(%)*	72.0 ± 18.8	73.7 ± 16.7	0.650^c^
*Postbronchodilator FEV*_*1*_*/FVC (%)*	63.9 ± 6.3	63.9 ± 6.4	0.985^b^
*GOLD stages*			0.457^a^
Stage I	9 (36.0)	38 (41.3)	
Stage II	13 (52.0)	48 (52.2)	
Stage III	3 (12.0)	4 (4.3)	
Stage IV	0 (0)	2 (2.2)	
*2020 GOLD groups*			0.745^a^
Group A	15 (60.0)	48 (52.2)	
Group B	9 (36.0)	37 (40.2)	
Group C	1 (4.0)	5 (5.4)	
Group D	0 (0)	2 (2.2)	

Data are expressed as mean ± standard deviation or as frequency (percentage). Statistically significant *P* values are presented in bold.

*BMI* body mass index, *CAT* COPD assessment test, *FEV*_*1*_ forced expiratory volume in 1 s, *FVC* forced vital capacity, *mMRC* modified Medical Research Council.

^a^χ^2^ test.

^b^Mann–Whitney *U* test.

^c^Independent-samples *t*-test.

COPD patients below the age of 55 years experienced wheezing and exacerbations more frequently than COPD patients older than 55 years. They were likely to have comorbidities, especially hypertension compared to older patients. Two-thirds of patients greater than or less than 55 years were newly diagnosed COPD patients.

### Comparisons of newly and previously diagnosed COPD patients

Newly diagnosed COPD patients represented 70.9% of the COPD group. The differences between demographic, clinical, and spirometric characteristics of newly and previously diagnosed COPD patients are presented in Table 3.

**Table 3:** Comparisons of demographic, clinical, and spirometric characteristics of newly vs. previously diagnosed COPD patients.

Characteristics	Newly diagnosed COPD patients (*n* = 83)	Previously known COPD patients (*n* = 34)	*P* value
*Age (years)*	59.6 ± 6.2	62.0 ± 4.7	**0.020**^**a**^
*Males*	63 (75.9)	31 (91.2)	**0.050**^**b**^
*BMI (kg/m*^*2*^*)*	27.5 ± 5.1	28/7 ± 4.8	0.210^a^
*Educational level*			0.600^b^
Elementary education	32 (38.6)	16 (47.1)	
Secondary education	45 (54.2)	15 (44.1)	
Tertiary education	6 (7.2)	3 (8.8)	
*Marital status*			0.300^b^
Not married	3 (3.6)	1 (2.9)	
Married	75 (90.4)	30 (88.2)	
Divorced	3 (3.6)	0 (0)	
Widowed	2 (2.4)	3 (8.8)	
*Current smokers*	64 (77.1)	24 (70.6)	0.450^b^
*Start smoking age (years)*	19.3 ± 8.0	16.9 ± 3.3	**0.020**^**a**^
*Pack-years of smoking*	46.7 ± 24.6	60.8 ± 25.5	**0.008**^**a**^
*Respiratory symptoms (yes)*	71 (85.5)	33 (97.1)	0.060^b^
Cough	44 (53.0)	23 (67.6)	0.140^b^
Sputum	36 (43.4)	27 (79.4)	<**0.001**^**b**^
Wheezing	42 (50.6)	24 (70.6)	**0.040**^**b**^
Dyspnea	54 (65.1)	27 (79.4)	0.120^b^
*mMRC* *≥* *2*	23 (27.7)	15 (44.1)	0.080^b^
*CAT* *≥* *10*	28 (33.7)	19 (55.9)	**0.020**^**b**^
*Exacerbations (yes)*	39 (47.0)	17 (50.0)	0.190^b^
One	36 (43.4)	13 (38.2)	0.600^b^
Two or more	3 (3.6)	4 (11.8)	**0.050**^**b**^
*Comorbidities (yes)*	50 (60.2)	20 (58.8)	0.150^b^
Hypertension	36 (43.4)	18 (52.9)	0.340^b^
Diabetes mellitus	13 (15.7)	5 (14.7)	0.890^b^
Cardiovascular disease	5 (6.0)	11 (32.3)	0.070^b^
Hyperlipidemia	27 (32.5)	10 (29.4)	0.740^b^
Depression	1 (1.2)	3 (8.8)	**0.030**^**b**^
Osteoporosis	3 (3.6)	1 (2.9)	1.000^b^
Prostatic hyperplasia	5 (6.0)	1 (2.9)	0.670^b^
*Postbronchodilator FEV*_*1*_ *(ml)*	2.26 ± 0.62	2.01 ± 0.66	**0.050**^**c**^
*Postbronchodilator FEV*_*1*_ *(%)*	76.4 ± 15.6	65.8 ± 18.2	**0.005**^**c**^
*Postbronchodilator FEV*_*1*_*/FVC*	65.6 ± 4.1	59.6 ± 8.6	**<0.001**^**a**^
*GOLD Stage I*	39 (47.0)	8 (23.5)	**0.019**^**b**^
*GOLD Stage II*	40 (48.2)	21 (62.0)	0.180^b^
*GOLD Stage III*	4 (4.9)	3 (8.8)	0.410^b^
*GOLD Stage IV*	0 (0)	2 (5.9)	0.080^b^
*2020 GOLD Group A*	52 (62.6)	12 (35.3)	**0.014**^**b**^
*2020 GOLD Group B*	28 (33.7)	17 (50.0)	0.162^b^
*2020 GOLD Group C*	3 (3.6)	3 (8.8)	0.350^b^
*2020 GOLD Group D*	0 (0)	2 (5.9)	0.080^b^

Data are expressed as mean ± standard deviation or as frequency (percentage). Statistically significant *P* values are presented in bold.

*BMI* body mass index, *CAT* COPD assessment test, *FEV*_*1*_ forced expiratory volume in 1 s, *FVC* forced vital capacity, *mMRC* modified Medical Research Council.

^a^Mann–Whitney *U* test.

^b^χ^2^ test.

^c^Independent-samples *t*-test.

Newly diagnosed COPD patients were significantly younger, were more likely to be female, had an older mean age of the first cigarette smoked, and smaller pack-year history than previously known COPD patients. Importantly, 85.5% of newly diagnosed COPD individuals reported at least one respiratory symptom, while 14.5% were asymptomatic. Moreover, we found that one-third of the newly diagnosed patients were highly symptomatic (mMRC ≥ 2 and CAT ≥ 10), and half of them had at least one exacerbation. Newly diagnosed and previously known COPD subjects have a similar percentage of cough and dyspnea. Patients with previously known COPD significantly more often suffered from coronary artery disease and depression than the newly diagnosed COPD group. Newly diagnosed patients had better lung function than known COPD patients.

The majority of newly diagnosed COPD patients were classified into GOLD Stages I and II. Approximately two-thirds of newly diagnosed COPD individuals were classified into GOLD COPD risk Group A, had fewer symptoms, and a low risk of exacerbation. Almost one-third of those patients had noteworthy respiratory symptoms and low risk of exacerbations classified into GOLD COPD risk Group B. Only patients with a previous diagnosis of COPD reached GOLD Stage IV, and GOLD COPD Group D.

A total of 25% of newly diagnosed patients were women classified from Stage I to Stage III. No differences in demographic and clinical characteristics were found between genders. Males had a lower level of lung function than women (forced expiratory volume in 1st second (FEV_1_)/forced vital capacity (FVC) ratio: 65.1 ± 4.43 vs. 67.5 ± 1.9, *P* < 0.018, respectively). Most of the patients who received the first diagnosis of COPD were married. No differences were found in the demographic characteristics of newly diagnosed COPD patients according to different GOLD COPD groups.

Notably, 20.5% (17/83) of the newly diagnosed patients were equal to or under the age of 55. In all, 52.9% (9/17) of those patients had already reached Stage II COPD and one patient reached Stage III COPD. A total of 23.5% (4/17) of these patients were assigned to Group B and the rest to Group A. No specific differences in demographic or clinical characteristics of newly diagnosed COPD patients aged less than 55 according to GOLD stages or GOLD COPD stages were found.

## Discussion

In the current study, a high COPD prevalence of 10.6% among 1100 current or ex-smokers aged 40–65 was estimated. In Greece, the reported COPD prevalence ranged from 8.6 to 18.4%^7^ (Table 4). The great variations observed in the prevalence rates were mainly attributed to the absence of comparable methodologies.

**Table 4:** Studies carried out in Greece estimating COPD prevalence (2000–2020)^7^.

Study	Year	Inclusion criteria	*N*	Males	Females	Average pys	Geographical distribution	COPD prevalence
Gourgoulianis et al.^50^	2000	>55 years >100 cigarettes	569	256	313	Not defined. Current smokers/non-smokers: 182/387	Central Greece	17.9%: urban areas 9.6%: rural areas
Tzanakis et al.^51^	2004	>35 years	888	475	413	Median: 26 pys	National	8.6%
Sichletidis et al.^52^	2005	21–80 years	6112	Not defined	Not defined	Pys in smokers: 29.3 ± 26.1 Pys in ex-smokers: 39.9 ± 30.5	Northern Greece	5.6%
Minas et al.^8^	2010	>30 years	1526	902	624	Pys in smokers: 47.27 ± 34.4 Pys in ex-smokers: 47.9 ± 39.4	Central Greece	18.4%
Mitsiki et al.^6^	2015	>40 years, known COPD	6125	4367	1758	Median: 40 pys (lower: 20, upper: 83.6)	National	18.2%
Kourlaba et al.^53^	2016	≥40 years, telephone survey	3414	Is not defined	Is not defined	Not defined	National	10.6%
Spyratos et al.^54^	2016	>40 years, smokers	3200	2096	1104	Median: 25 pys	Thessaloniki	10.7%
Stafyla et al.^12^	2018	≥40 years, smokers	186	127	59	Median: 50 ± 37.8 pys	Central Greece	17.8%

*pys* pack-years.

Conversely, our results differ from other national studies of those exposed to tobacco that found higher COPD prevalence^13^. The variations observed in the prevalence rates were mainly due to differences in age and sex distribution and other population characteristics evaluated in each study. More specifically, we limited the population to those 40–65 years of age, given that global estimates have reported that although COPD rises significantly in the sixth decade and beyond, COPD is not seen any longer as a disease of the elderly, as a significant proportion of patients (up to 40%) with COPD are aged less than 65 years^14^. We focused on the concept of early COPD diagnosis, which is an emerging area for research.

A prior study of our team has noted that the underdiagnosis and missed diagnosis of COPD among the primary care smoking population is an old problem that persists in Greece^12^. A total of 7.5% of the study population received a diagnosis of COPD for the first time. Of the 117 patients finally diagnosed after studying 1100 people, 83 were diagnosed for the first time, representing a rate of 70.94% of underdiagnosis. Similarly, a previous post-economic crisis study conducted in ten primary healthcare centers of Central Greece during a 7-month period, including 186 current smokers or ex-smokers aged ≥40 years, demonstrated a prevalence rate of first**-**time diagnosed COPD of 7.5%^12^. Another pre-economic crisis study (conducted from January 2006 to June 2007) included 15 primary healthcare centers in central Greece, with a total of 1526 subjects aged over 30 years, documented a higher prevalence rate of new COPD cases of 18.4%^8^. The leading causes of COPD underdiagnosis and misdiagnosis are attributable to the lack of training and feedback for diagnostic spirometry, the lack of adoption of GOLD guidelines into routine clinical practice, and the improper symptom assessment by healthcare professionals^15,16^. Moreover, as our research findings highlight that decreased patients’ awareness contributes to COPD underdiagnosis.

A total of 20.7% of all COPD patients and 25% of newly diagnosed patients were women. It has been reported that approximately 20% of the smoking population worldwide are women^16^. Owing to the rising prevalence of COPD in women, increasing mortality risk for females with COPD is expected. The relative risk of death from COPD is currently nearly equal for males and females^17^. There is increasing evidence that in COPD pathogenesis, sex differences describe biology-linked differences between men and women, and sex biology has been associated with increased risk for COPD in women^18,19^. Estrogen induces lung differentiation and maturation; however, by the age of menopause, there is a considerable loss of lung function associated with smoking and estrogen and progesterone decline^18,19^. Our results support previous findings claimed that women with COPD reported smoking fewer cigarettes than men, had an older age at starting smoking, but experienced worse symptoms^19^. Women manifest more severe COPD symptoms across life course^19^. Consistent with the literature, this research found that females were more depressed than males^20^. COPD is a severe treatment-resistant pulmonary disease with varying impacts on the patient’s general physical condition, functioning, and quality of life. Depressive symptoms are common in patients affected by COPD, even when their disease is mild^21^. Female patients appear to be more susceptible to psychological risks. Although being diagnosed with depression can be hard, not receiving a definite diagnosis can be even more difficult to deal with^21^.

One of the most interesting findings of our study was that two out of ten patients among the COPD population were below 55 years. The vast majority of them were newly diagnosed COPD patients. Severe early-onset COPD is an interesting phenotype defined as COPD development in individuals younger than 50–55 years and often with a light pack-year smoking history^22,23^. There is evidence that these patients progress quickly^24^. Half of the newly diagnosed patients below 55 years had already reached Stage II COPD in our study. No information is available on the true impact of COPD of recent onset mainly because COPD has been previously considered a disease of the elderly^25^. The global prevalence of COPD in smokers or ex-smokers aged <50 years has been roughly estimated at 4.1% (95% CI 2.9–5.4%)^25,26^.

A few data regarding the age distribution of newly diagnosed COPD patients in Central Greece reported a higher mean age of new COPD diagnosis between 65 and 69 years^8,12^. In this study, we found that newly diagnosed COPD patients were significantly younger (59.6 ± 6.2 vs. 62.0 ± 4.7), had an older mean age of the first cigarette smoked, a smaller pack-year history than previously known COPD patients. Therefore, our findings supported that COPD patients were first diagnosed at a younger age than the national average of COPD diagnosis. COPD is a disease of accelerated aging. However, COPD is not considered any longer as an illness of the aged, as it can also affect younger adults under 65 years old^3^. Furthermore, COPD risks may already occur in adolescents, on the assumption that some are born with reduced pulmonary function and that infection and exposure to toxins at an early age may greatly reduce their pulmonary capacity^3^.

Ever-smokers with respiratory symptoms should have more careful health care. Importantly, 85.5% of newly diagnosed COPD individuals reported at least one respiratory symptom, while approximately two-thirds of newly diagnosed COPD individuals were classified into GOLD COPD risk Group A, had fewer symptoms, and a low risk of exacerbation. Indeed, many people with COPD symptoms do not meet the criteria for diagnosis according to lung-function tests indicating that they are healthy^27,28^. However, it has been documented that nearly 40% of the people who did not meet the definition of COPD when they joined an epidemiological study had late-stage disease 5 years later^29–31^.

This study found that one-third of the newly diagnosed patients were highly symptomatic (mMRC ≥ 2 and CAT ≥ 10), and half of them had at least one exacerbation. Despite the burden of symptoms, they did not seek medical help, as they felt COPD symptoms as part of their daily smoking routine or due to aging. These data indicate that COPD awareness is poor among smokers, and strategies to increase COPD awareness should be developed^32–34^. The smoking population underestimates their respiratory symptoms; however, exercise activity is many times reduced. Therefore, to detect new COPD cases, questions related to respiratory vulnerability and deconditioning are required^27,35^. Besides, recent studies showed that exacerbations are frequent in smokers, even they had normal spirometry tests. Another recent study showed that the presence of symptoms in ever-smokers with preserved pulmonary function is correlated with more exacerbations, activity limitation, and greater airway-wall thickening with or without emphysema^28,36,37^.

On the other hand, a significant percentage of 14.5% of the newly diagnosed COPD population was asymptomatic. This finding also accords with earlier observations, which showed a high prevalence of COPD among smokers with no symptoms^9,38^. Even among subjects with severe airflow obstruction, a substantial proportion did not report symptoms^39,40^. About 40% of those in the GOLD severe category denied being breathless^39^. Therefore, a mass screening on all smokers to detect COPD is suggested^9,38^. This case-finding strategy of COPD detection by screening for symptoms that patients may not themselves perceive is very important in primary care^38^. In addition, it is of high importance to screen smokers who had minimal or no symptoms or limited activity, regardless of their age, gender, or how long they have been smoking, for COPD to establish an early diagnosis for COPD patients to stop disease progression and improve the quality of life via the motivation for physical activity, smoking cessation, and potential pharmacological treatment^38^.

Another important finding of this study was that 60% of newly diagnosed COPD patients had at least one comorbidity. Tobacco smoking is a risk factor for many comorbidities, as well as for COPD. Systemic inflammation appeared to be shared across multiple chronic diseases^41,42^. Besides, we found that cardiovascular disease and depression were more likely to be developed in known COPD patients with higher smoking intensity than newly diagnosed patients. Certainly, people with COPD have a higher risk of cardiovascular disease^43^. COPD severity and duration have been correlated with increased risk of cardiovascular disease or depression^43,44^. On the other hand, patients with pre-existing depression or developed depression after COPD diagnosis were more likely to suffer from heightened COPD symptoms, such as increased breathlessness, reduced exercise tolerance, and hopelessness^44^.

The findings in this study are subject to a major limitation. The study was carried out on a local scale, which reduces the generalizability of the results. Moreover, this study limits the study population to those 40–65 years of age. It is expected that prevalence will increase in older age groups as a function of age and cumulative pack-years.

This primary care COPD screening program was implemented in ever-smokers younger than 65, and an increased prevalence rate of new COPD diagnosis comes to light. Women with COPD reported smoking fewer cigarettes than men, had an older age at starting smoking, but experienced worse respiratory and depressive symptoms. COPD is not considered any longer as an illness of the aged as it can also afflict the younger population. The findings clearly indicate that most newly diagnosed COPD patients reported a significant burden of symptoms without seeking medical help and finally diagnosed at a moderate stage of the disease. Approximately half of them had at least one exacerbation during the last year and complained of comorbid diseases. The underestimation of respiratory symptoms by smokers is a thorn in COPD diagnosis. A reasonable approach to tackle this issue could be to screen all smokers who report minimal or limited activity or even no respiratory symptoms, regardless of their age, gender, or how long they have been smoking, for COPD, and to implement smoking cessation counseling to control the disease’s progressiveness. Strategies to increase COPD awareness and change the natural history of COPD should be developed. The rule is that there are no rules in COPD; thus, primary health care has a crucial role in the early detection of COPD among unsuspecting smokers. The challenge is to find tactics accelerating the early detection of disease in primary care.

## Methods

A 2-year (January 2018–December 2019) spirometry surveillance program was conducted in 22 primary healthcare centers in rural and semirural areas in Thessaly, Central Greece. All current smokers or ex-smokers resided near a primary healthcare setting, aged 40–65 years, and were willing to participate in a spirometry surveillance program, were included in the study. Smoking status was categorized into: (1) former smokers included adults who have smoked at least 100 cigarettes in their lifetime but who had quit smoking for at least a year and (2) current smokers included adults who have smoked 100 cigarettes in their lifetime and currently smoke cigarettes daily or nondaily. Smoking status for all subjects was measured in pack-years of cigarette smoking (pys). Exclusion criteria included no smoking history, a history of lower or upper respiratory infection or antibiotic treatment within the last month, recent eye surgery, an operative thoracic or abdominal procedure, or acute myocardial infarction within the last 3 months, and inability to perform spirometry. People with a lower or upper respiratory infection or antibiotic treatment within the last month were temporarily excluded from the study and scheduled for inclusion some weeks later. Ethical approval for this study was obtained from 5th Dypethessaly (Regional Health Authority of Thessaly & Sterea Ellada, Greek Ministry of Health) (Ref no: 20227/April 25, 2018 and Ref no: 2163/January 9, 2019 from 5th Dypethessaly). All subjects provided written informed consent.

### Study design

Pre- and postbronchodilator spirometry tests were performed to establish the diagnosis of COPD, using portable spirometers. COPD patients filled out a questionnaire including questions on demographics, smoking status, occupational exposure, chronic respiratory symptoms (cough, sputum, wheezing, and dyspnea), history of respiratory infections, comorbidities, and exacerbations within the last 2 years. In addition, the CAT and the mMRC were completed by COPD participants to measure the impact of COPD on their life.

### Spirometry procedure

Spirometry test was performed with a dry spirometer (Spirolab FCC ID: TUK-MIR045) according to American Thoracic Society recommendations^45^. Calibration checks were performed every morning before the beginning of the spirometry program. Spirometry testing was performed by eight physicians and two nurses who had undergone a special training program. Forced expiratory maneuvers were repeated until three acceptable reproducible tests were obtained and the best FEV_1_, FVC, and FEV_1_/FVC ratio were recorded. The spirometric reference values used were those proposed by the ERS statement^46^. An acceptable maneuver was defined using the previously described criteria^47^.

### Diagnosis of COPD

COPD diagnosis was established with the presence of a postbronchodilator FEV_1_/FVC ratio less than 0.70 following GOLD guidelines^48,49^. All patients under inhaled medication were instructed to avoid their inhalers for 12 or 24 h depending on the drug formulation (for instance, SABA ≥ 4 h, LABA ≥ 15 h, LAMA ≥ 12 or 24 h) before spirometry. A bronchodilator reversibility test with 400 µg of salbutamol was performed in all patients with a baseline prebronchodilator obstructive spirometry. A positive response to bronchodilators was defined as an increase in the FEV_1_ of 200 ml and 12% from the prebronchodilator value (baseline value). All participants with a positive bronchodilator response were excluded from the analyses. Classification of COPD was based on the postbronchodilator FEV_1_ (%) according to GOLD guidelines (Stage I/mild COPD, FEV_1_ ≥ 80%; Stage II/moderate COPD, 50% ≤ FEV_1_ < 80%; Stage III/severe COPD, 30% ≤ FEV_1_ < 50%; Stage IV/very severe COPD, FEV_1_ ≤ 30% or FEV_1_ < 50% with respiratory failure)^49^. Patients with COPD were also allocated into four groups by GOLD (GOLD Groups A, B, C, and D) according to the 2020 GOLD guidelines^49^.

### Statistical analyses

Demographic data were presented as mean ± standard deviation. Categorical variables are presented as percentages. Differences in numerical variables were evaluated with independent-samples *t*-test or Mann–Whitney *U* test and Tukey’s multiple comparisons test for normally and skewed data, respectively, whereas comparisons of proportions were performed using χ^2^ test with applying the Bonferroni correction. *P* values < 0.05 were considered statistically significant. The results were analyzed statistically with SPSS 26, and graphs were made using PRISM 8.

### Reporting summary

Further information on research design is available in the Nature Research Reporting Summary linked to this article.

## Supplementary information

Reporting Summary

## Data Availability

The data that support the findings of this study are available on request from the corresponding authors, O.S.K. and E.G. The data are not publicly available due to restrictions, e.g., their containing information that could compromise the privacy of research participants.
